# Supplementary figures and images for: Larazotide Acetate Protects the Intestinal Mucosal Barrier from Anoxia/Reoxygenation Injury via Various Cellular Mechanisms
Source: Biomedicines. 2025 Oct 12;13(10):2483. doi: 10.3390/biomedicines13102483 (PMC12561949; doi:10.3390/biomedicines13102483)

Supplementary figure 1.

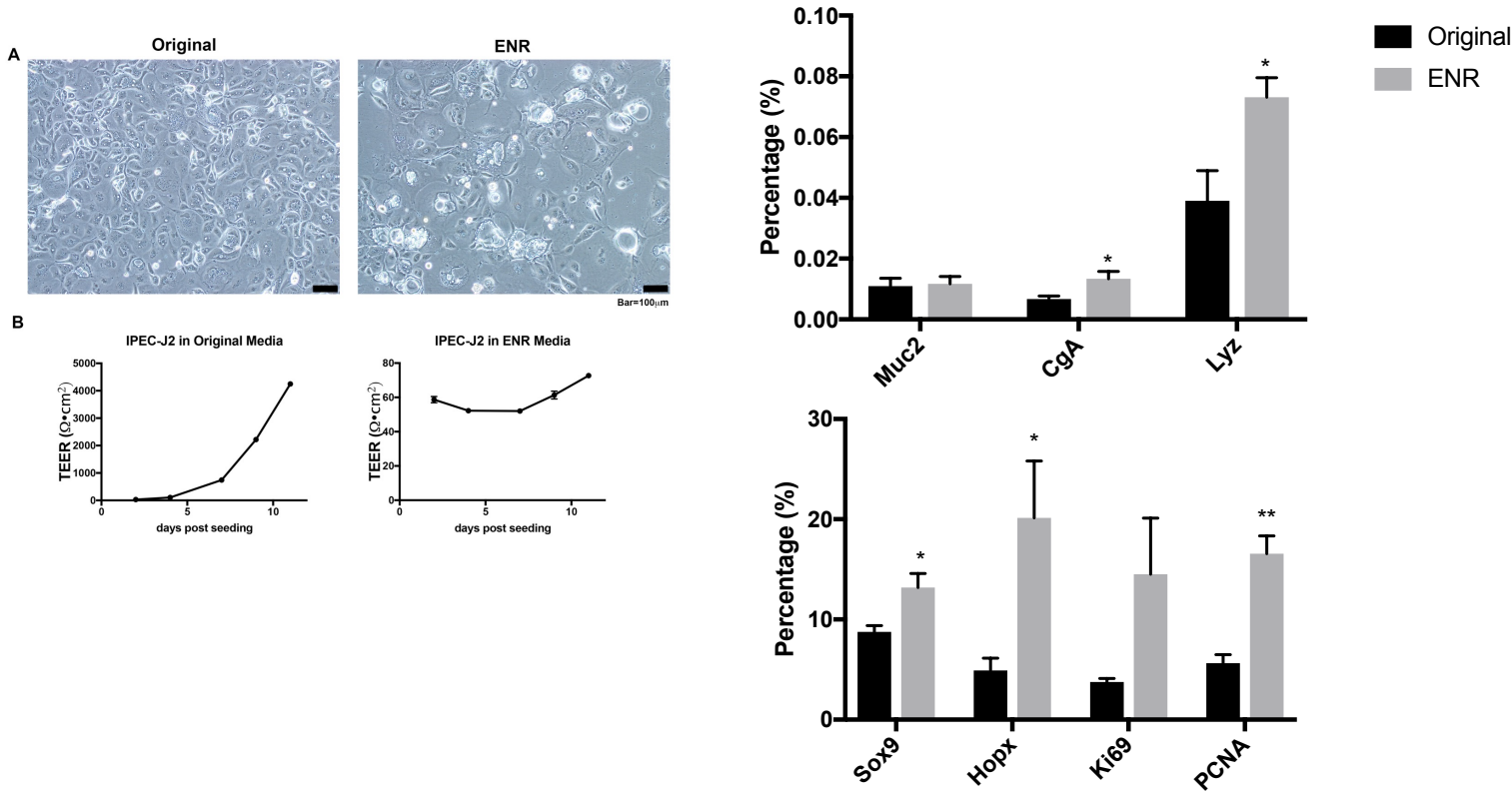

Supplement: Supplementary file 1 [file biomedicines-13-02483-s001.zip › Supp. Fig. 1.pdf]
